# Comparing outcomes of robotic-assisted radical prostatectomy by specialists and trainees using a modular training approach

**DOI:** 10.1007/s11701-025-02277-6

**Published:** 2025-05-13

**Authors:** Zoe Williams, Jeremy Saad, Femi E. Ayeni, Henry Wang, Wenjie Zhong, Rasha Gendy, Mohan Arianayagam, Bertram Canagasingham, Ahmed Goolam, Nicola Jeffery, Jonathan Kam, Mohamed Khadra, Raymond Ko, Nicholas Mehan, Celalettin Varol, Isaac Thangasamy

**Affiliations:** 1 Nepean Urology Research Group, Sydney, Australia; 2 Department of Urology, Nepean Hospital, Derby St, Kingswood, Sydney, NSW Australia; 3 Sydney Medical School, Faculty of Medicine and Health, University of Sydney, Sydney, Australia; 4 Faculty of Medicine, Health, and Human Sciences, Macquarie University, Sydney, Australia

**Keywords:** Robotic prostatectomy, Surgical training, Robotic training, Modular training, Surgical education, Dual console

## Abstract

Robotic-assisted laparoscopic prostatectomy (RALP) is the dominant surgical approach for prostate cancer worldwide. The steep learning curve in robotic surgery is eased by modular training and the da Vinci Surgical System^©^ dual console, where supervisors can assume control of the robot from a secondary console if required. Here we evaluate the safety of robotic training by comparing pathological and peri-operative outcomes of RALPs performed predominantly by urology trainees supported by a modular training approach and dual console supervision with RALPs performed predominantly by specialist robotic surgeons. This prospective cohort study examines RALPs performed at a tertiary robotic training centre in Australia between February 2017 and August 2018. Each case was divided into 13 steps from port placement to specimen retrieval. A case was considered a ‘trainee-lead case’ if the trainee completed more than 75% of the operative steps. We compared patient demographics, operative parameters, peri-operative outcomes, and pathological outcomes between groups. Differences between groups were measured using Fisher’s exact test for categorical data and the unpaired Student’s t-test for continuous data. Of 126 cases in this study, 39 (31%) were trainee-led cases and 87 (69%) were specialist lead cases. There was no significant difference in operative or pathological outcomes between trainee-lead cases and specialist-lead cases. Our results compared favourably with local and international benchmarks. RALP performed by trainees using a modular training approach and supported by the dual console can have equivalent peri-operative and pathological outcomes to specialist-led cases. This is achieved by graded progression and dual console supervision.

**Supplementary Information:**

The online version contains supplementary material available at 10.1007/s11701-025-02277-6.

## Introduction

Prostate cancer is the second-most diagnosed cancer worldwide [1] and the fifth-leading cause of cancer-related mortality [2]. Treatment modalities include surgery, radiotherapy, chemotherapy, and hormonal therapy [3]. Treatment recommendations are informed by goals of therapy, disease profile, comorbidities, and patient preferences. Patients with localised or locally advanced prostate cancer are counselled on both surgical intervention and radiation therapy [4].

Surgical techniques for prostatectomy include open radical prostatectomy (ORP), laparoscopic radical prostatectomy (LRP), and robotic-assisted laparoscopic prostatectomy (RALP) [5]. Traditionally, ORP was most commonly performed. However, with the emergence of the da Vinci Surgical System^©^ (Intuitive Surgical, CA, USA), first used for prostatectomy in 2001, RALP is now the dominant surgical approach [6]. Worldwide, 62% of prostatectomies performed are RALPs, 39% LRPs, and 1% ORPs [7–10]. In Australia, there has been a steady shift to RALP and it is now the dominant approach in metropolitan centres [11].

Unlike familiar laparoscopic and open techniques, robotic surgery poses new challenges to trainees [12]. Surgical robots are designed with unfamiliar operative controls requiring new dexterity. They also lack tactile feedback and use a three-dimensional visual interface on a separate console. These challenges are difficult to tackle for the first time intraoperatively. Further, the appropriate priority of patient safety frequently results in specialists assuming control of the robot from trainees, which reduces trainees’ time on the console. In RALP, two specific learning curves are described—a ‘basic learning curve’ to achieve comparable perioperative outcomes, such as appropriate complication rates and operative time, and an ‘advanced learning curve’ to achieve appropriate pathological and functional outcomes [13, 14]***.***

Emerging approaches to robotic surgical training include modular training programs and dual console support [12]. Modular training is an educational method of graded proficiency-based training. It involves stepwise progression through key operative steps of increasing difficulty [15]. Compared with conventional procedural training, proficiency-based approaches improve performance by reducing intra-operative errors and operative time [16]. Several modular training pathways have been developed for RALP [14, 15, 17]. They involve combinations of e-learning videos and simulation training in dry-labs, wet-labs, and virtual reality contexts. Trainees complete modules to achieve RALP competencies in a stepwise manner within a structured educational framework.

The da Vinci Surgical System^©^ facilitates modular training in RALP. The ‘da Vinci SimNow^TM^’ generates a virtual, interactive three-dimensional surgical experience where key procedural steps are simulated in compartmentalised exercises [18]. The dual console system also supports modular training in RALP by facilitating one-on-one training from specialists supervising from a secondary console. This learning system is designed to prioritise the acquisition of skills without compromising patient outcomes [19].

Currently in Australia there is no structured urological robotic surgery curriculum. In this work, we developed a modular training program for RALP at a high-volume urological robotic centre in Sydney, Australia. Our modular training program involved undertaking da Vinci Surgical System^©^ simulation exercises and receiving intra-operative specialist instruction through the da Vinci Surgical System^©^ dual console. Here, we assess the safety and efficacy of modular training and dual console support in RALP. We compare peri-operative and pathological outcomes of RALPs performed predominantly by specialist urologists with those performed predominantly by urology trainees who undertook the modular training program. This assessment will determine patient safety, procedural quality, and teaching outcomes in RALP using a modular training approach.

## Methods

This study reviews prospective data from the Nepean Hospital Urology Department in Sydney, Australia from February 2017 to August 2018. Ethics approval was obtained via the Nepean Blue Mountains Local Health District Human Research and Ethics Committee (approval number: Study 17–53A).

### Case selection

Patients who underwent RALPs with the da Vinci Surgical System^©^ between February 2017 and August 2018 were included in this study. Eight RALP specialists and four fellowship level or penultimate year urology trainees (two in 2017 and two in 2018) performed the RALPs in this study. Urology trainees completed introductory da Vinci Surgical System^©^ in-built training modules relevant to RALP prior to participating in the corresponding steps in RALP cases.

Case selection was determined by trainee experience and procedural difficulty. As per the modular training approach, trainees achieved stepwise technical competency in RALP operative steps by completing relevant simulation training modules and then performing corresponding steps intraoperatively, supervised at all times by a specialist on the dual console. As trainees’ technical competency developed throughout the study, trainees performed a greater number of operative steps independently. Mastery of each operative step by trainees was determined by individual performance rather than case numbers. Cases were allocated to the trainee-lead group or the specialist-lead group at the conclusion of the case according to the proportion of the procedure completed by the trainee. Given case allocation was performed at the conclusion of the procedure, patients were not informed of the proportion of steps performed by the trainee unless they explicitly enquired. This is consistent with standard operative practice for comparable procedures at our institution.

### Operative steps

In every RALP, the following key operative steps were performed by the urology trainee or the specialist urologist:

1. Robot docking and port placement
2. Retropubic dissection
3. Endopelvic fascia incision
4. Anterior bladder neck transection
5. Posterior bladder neck transection
6. Dissection of seminal vesicles and vas deferens
7. Denonvillier dissection
8. Lateral pedicle dissection
9. Ligation of dorsal venous plexus
10. Apical dissection and urethral division
11. ROCCO stitch
12. Urethrovesical anastomosis
13. Specimen retrieval +/- pelvic lymph node dissection

If the trainee completed more than 75% of the operative steps (10 of 13), the case was classified as a ‘trainee-lead case’.

### Data collection

At the completion of each RALP, the urology trainee involved completed the data collection form (Appendix 1). Peri-operative parameters including age, body mass index (BMI), pre-operative haemoglobin level, pre-operative prostate specific antigen level (PSA), prostate volume, International Society of Urological Pathology (ISUP) grade group, and pathological stage were recorded. Operative parameters included total console time, estimated intra-operative blood loss, blood transfusion requirement, intra-operative adverse events, post-operative intensive care requirements, length of hospital stay (LOS), duration of urethral catheterisation, and the degree of pelvic lymph node dissection (PLND) performed. Thirty-day Clavien–Dindo (CD) complications were also recorded. Pathological outcomes collected were surgical margin status and detectability of PSA at 6 and 12 weeks post-operatively.

### Data storage and analysis

A data manager and a research assistant entered the information collected from the data collection form into a master Microsoft Excel (Microsoft Corporation, WA, USA) sheet. Statistical analysis was performed using GraphPad Prism 10 (Dotmatics, MA, USA). Continuous variables were reported as mean and standard deviation for normally distributed parameters and median and interquartile range for non-normally distributed parameters. Categorical variables were reported as frequency data. Tests of normality were performed as required using Shapiro–Wilk and Kolmogorov–Smirnov test. Differences between groups were measured using the Fisher’s exact test for categorical data and unpaired student t-test or Mann-Whitney test for continuous data. Significant level was accepted at *p *< 0.05.

## Results

Of 126 cases included in the study, 31% (*n* = 39) were performed predominantly by urology trainees and 69% (n=87) were performed predominantly by specialist urologists. Of the 87 cases performed predominantly by specialist urologists, the number of steps completed by a urology trainee ranged from 0–5 (0–38%) of the 13 operative steps. Based on our sample size with medium effect size, our study power is 73%.

Table 1 demonstrates patient demographics and disease characteristics of patients who underwent RALPs in this study. There was no significant difference in patient demographics (age, BMI), pre-operative parameters (PSA level, prostate volume, haemoglobin level), or disease characteristics (ISUP grade group, pathological stage) between trainee-lead cases and specialist-lead cases.

**Table 1:** Patient demographics and prostate cancer disease profile of patients undergoing RALP

	Trainee-lead cases	Specialist-lead cases	P-value
Number of patients	39 (31.0%)	87 (69.0%)	
Age (years)	63.4 (8.0)	63.5 (7.2)	0.9551
BMI	28.6 (5.0)	28.2 (4.6)	0.7138
Pre-operative PSA (ng/mL)	6.4 [4.2]	6.2 [4.15]	0.9659
Pre-operative Prostate Volume (cc)	30 [15.5]	37.5 [24.13]	0.2965
Pre-operative Haemoglobin (g/L)	146.3 (20.6)	145.9 (16.6)	0.9122
ISUP Grade Group			0.6361
1	2 (5.1%)	6 (6.9%)	
2	21 (53.8%)	40 (46.0%)	
3	9 (23.1%)	16 (18.4%)	
4	5 (12.8%)	13 (14.9%)	
5	2 (5.1%)	12 (13.8%)	
Pathological Stage			0.3084
T2	16 (41.0%)	37 (42.5%)	
T3a	22 (56.4%)	39 (44.8%)	
T3b	1 (2.6%)	10 (11.5%)	
T4	0	1 (1.1%)	

Data are represented as mean and (standard deviation), median and [interquartile range], or frequency and percentage

Table 2 demonstrates operative parameters and peri-operative outcomes of patients in this study. There was no significant difference in operative parameters (robotic console time and frequency and degree of PLND) and peri-operative outcomes (intra-operative adverse events, estimated total blood loss, blood transfusion requirement, post-operative intensive care unit (ICU) admissions, duration of hospital admission, readmissions post discharge, duration of indwelling catheter use, and frequency of CD complications). Intra-operative adverse events occurred in two cases. These events were a caecal injury in a specialist-lead case (*n* = 1) and intra-operative blood loss of 1.5 litres in a trainee-lead case (*n* = 1). One patient in the specialist-lead group required ICU admission post-operatively. This patient represented 3 weeks post-operatively with bilateral segmental and subsegmental pulmonary emboli and required intensive care.

**Table 2:** Operative characteristics and peri-operative outcomes of RALP

		Trainee-lead cases	Specialist-lead cases	P-value
Console Time (mins)		172.4 (57.9)	152.3 (60.9)	0.1489
PLND Status				0.5179
Not performed		3 (7.7%)	5 (5.7%)	
LPLND negative		31 (79.5%)	62 (71.3%)	
LPLND positive		1 (2.6%)	1 (1.1%)	
EPLND negative		4 (10.3%)	16 (18.4%)	
EPLND positive		0	3 (3.4%)	
Intra-operative Adverse Events		1 (2.6%)	1 (1.1%)	0.5250
Estimated Total Blood Loss (mL)		400 [300]	350 [400]	0.4910
Blood Transfusion		1 (2.6%)	1 (1.1%)	0.5250
Post-operative ICU Admission		0	1 (1.1%)	> 0.9999
Duration of Hospital Stay (days)		1 [1]	1 [1]	0.9990
Readmissions		3 (7.7%)	14 (16.1%)	0.2657
Post-operative CD Complications				0.2833
Grade 1		5 (12.8%)	7 (8.0%)	
Grade 2		1 (2.6%)	10 (11.5%)	
Grade 3a		0	0	
Grade 3b		1 (2.6%)	3 (3.4%)	
Grade 4a		0	2 (2.3%)	
Grade 4b		0	0	
Grade 5		0	0	
Indwelling Catheter Duration (days)		10 [1]	9 [1]	0.9569

Data are represented as mean and (standard deviation), median and [interquartile range]. or frequency and percentage

*LPLND* limited pelvic lymph node dissection, *EPLND* extensive pelvic lymph node dissection

The overall post-operative complication rate was 23%, of which 9.5% were CD1, 8.7% CD2, 3.2% CD3b, and 1.6% CD4a complications. There were no CD5 complications. Grade 1–2 complications in the trainee-lead group consisted of constipation, atelectasis, suprapubic pain, wound site ooze (*n* = 2), and low-grade fever. The grade 3b complication in the trainee-lead group was an infected lymphocele requiring intravenous antibiotics. Grade 1–2 complications in the specialist-lead group consisted of constipation (*n* = 2), abdominal pain (*n* = 3), nausea, reduced mobility, haematuria (*n* = 2), hypotension, syncope, urinary tract infection (*n* = 2), seroma, anaemia, blocked indwelling catheter, and bladder spasm. Grade 3b complications included a wound infection requiring drainage and anastomotic leak (*n* = 2), one with urosepsis. Grade 4a complications in the specialist-lead group included right middle cerebral infarct and pancreatitis with systemic inflammatory response syndrome.

Table 3 demonstrates the pathological outcomes of trainee-lead and specialist-lead RALPs. One specialist-lead case was pathological stage T4 and was excluded from pathological analysis. The positive surgical margin rate was 26.4% (11.3% T2 disease and 37.5% T3 disease). There was no significant difference in the rate of positive surgical margins between trainee-lead and specialist-lead cases. PSA remained undetectable in the majority of patients at 6 weeks (94.4%) and at 12 weeks (94.4%).

**Table 3:** Pathological outcomes of RALP

		Trainee-lead cases	Specialist-lead cases	P-value
Surgical Margins				
Negative		30 (76.9%)	62 (71.3%)	0.6643
Positive		9 (23.1%)	24 (27.9%)	
T2 total cases		16 (41.0%)	37 (43.0%)	0.6548
T3 total cases		23 (59.0%)	49 (57.0%)	
T2 negative surgical margins		15 (93.7%)	32 (86.5%)	0.7992
T2 positive surgical margins		1 (6.3%)	5 (13.5%)	
T3 negative surgical margins		15 (65.2%)	30 (61.2%)	
T3 positive surgical margins		8 (34.8%)	19 (38.8%)	
Undetectable PSA				
6 weeks post-operative		36 (92.3%)	82 (95.3%)	0.6764
12 weeks post-operative		37 (94.9%)	81 (94.2%)	> 0.9999

Data are represented as frequency and percentage

## Discussion

The integration of robotic systems in urology has surged in recent years [20]. Exposure of urology trainees to robotic surgery is becoming increasingly important. Our study compared the outcomes of trainee-lead RALP with specialist-lead RALP at a high-volume robotic centre in Australia to assess whether a modular training approach supported by specialist supervision through the dual console can provide high-quality robotic training without compromising patient outcomes. Our study of 126 RALPs found no significant difference in operative or pathological outcomes between trainee-lead and specialist-lead cases. We thereby demonstrate that stepwise progression through modular training, assisted by supervision from a dual console, is safe in RALP and does not compromise patient outcomes.

In line with our modular training approach of graded achievement of competency of RALP steps, case selection in our study was informed by trainee experience and operative skill. Introductory skills such as robot docking, port placement, and retropubic dissection were mastered prior to more technically difficult steps. Mastering the challenging RALP steps of apical dissection and nerve sparing was assisted by modular training and the dual console. Trainees worked through the proficiency-based system at their own pace, first practising skills in da Vinci simulations and then later intra-operatively with supervision from the dual console. The modular training and dual console system provided a safe training environment by allowing the specialist to assume control at a moment’s notice in the event of a pending complication or error. Despite no statistically significant difference in pre-operative parameters and disease profile between groups in our study, specialists likely undertook the more technically challenging cases, as steps in challenging patients that were above the trainee’s competency were not recorded as ‘trainee-completed’. This approach reflects real-world practice of appropriate case selection in a surgical training environment.

The peri-operative and pathological outcomes of trainee-lead RALP at Nepean Hospital are comparable with both local and international benchmarks [7–9, 21] (Table 4). Our data compares favourably in terms of case volume, pre-operative parameters, estimated total blood loss, Clavien–Dindo complications, and surgical margins. RALPs in other studies were performed by robotic surgeons with significant experience, usually a 2-year robotic fellowship and at least 200 RALPs performed [6–9, 19]. This experience is comparable to the experience of specialists in our study.

**Table 4:** Summary of representative studies worldwide assessing RALP outcomes

	Cathcart et al. (2011)[8]	Murphy et al. (2008)[9]	Yaxley et al. (2016)[21]	Alemozaffar et al. (2015)[10]	Laird et al. (2015)[7]	Current study (2024)
Country	AUS, NZ	AUS	AUS	US	UK	AUS
Study type	SR (n = 28)	Cohort	RCT	Cohort	Cohort	Cohort
Year RALPs performed	1980–2010	2003–2006	2010–2014	2000–2010	2011–2012	2017–2018
Number of patients	1783	400	157	282	362	126
Age (years)	N/R	60.2 (6.0)	59.6 (6.6)	67.2	61.5	63.5 [7.5]
BMI	N/R	27.2 (3.3)	N/R	26.4	N/R	28.7 (4.9)
PSA (ng/ml)	N/R	7.0	7.4 (4.1)	5.0	N/R	6.3 (4.0)
Total operative time (mins)	Range 148–251	186 (49)	246 (55)	N/R	N/R	212 (0.1)
Estimated total blood loss (mL)	Range 281–565	N/R	444 (294)	207	N/R	400 [400]
Blood transfusions	Range 0.9–3.0%	10 (2.5%)	1 (1%)	4.3%	N/R	2 (1.6%)
Clavien-Dindo Complication Rate	16%	16%	5%	N/R	10.3%	23.0%
Grade 1-2	N/R	11%	4%	N/R	9.2%	18.3%
Grade 3	5%	5%	1%	N/R	10%	3.2%
Grade 4	0.8%	0	0	N/R	1%	1.6%
Grade 5	0	0	0	N/R	0	0
IDC duration (days)	6.3	8.2 (3.1)	8.2 (3.6)	N/R	N/R	9 [1]
Duration of Hospital stay (days)	2.9 (range 1.1–5.5)	3.1 (1.4)	1.55 (2.6)	1.8	2.5	1 [1]
Positive Surgical Margins	16.7%	19.2%	15%	24.5%	N/R	26.2%
T2	N/R	9.6%	3%	N/R	13%	11.3%
T3	N/R	42.3%	11%	N/R	38.8%	37.5%

Data are represented as mean and standard deviation or frequency and percentage where available

*AUS* Australia, *NZ* New Zealand, *UK* United Kingdom, *US* United states, *SR* Systematic review, *RCT* Randomised controlled trial, *N/R* Not reported

When comparing our results with similar international studies, we must note that few of these international studies were randomised or well-designed comparative studies. Most were cohort studies with a high degree of heterogeneity [7, 9, 10]. Further, few studies primarily examined RALP outcomes. The larger studies showed marked improvement in outcomes when accounting for surgeon experience level. Cathcart et al. concluded that complication rates reduced after performing between 170 and 200 RALPs. In addition, many studies noted difficulties in correlating complication rates due to the variability in grading methods. The Clavien–Dindo system was not used consistently to score complication rates [8, 10], which most likely accounts for a higher grade 1–2 complication rate in our study.

Several RALP training programs have been developed internationally and incorporate modular training. Lovegrove et al. developed a 17-step modular training pathway for RALP with stepwise progression according to procedure step difficulty [15]. The European Association of Urology Robotic Training Curriculum incorporates e-learning modules, structured simulation-based training in virtual reality, synthetic, animal and cadaver platforms, and supervised modular training [17]. In a pilot study of this extensive training program, all participating fellows achieved independent competency in all RALP steps [22]. In a study by Schiavina et al., a 3-month intensive structured modular training program shortened the RALP learning curve to 20–30 cases to achieve shortened operative time and complications rates, and 90 cases to achieve optimal functional outcomes [14].

Modular training in RALP was supported by real-time guidance and intervention from specialists from a dual console in our study. Crucially, this setup permits a seamless transfer of device control with minimal tissue manipulation between operators [19]. A recent qualitative study by Green et al. recommended the dual console in robotic training as it enables competency-based learning and graded autonomy with appropriate verbal guidance [23]. Morgan et al. explored the benefits of the dual console system in RALP training. In this study trainees performed progressively more operative steps but without a structured modular training framework. The use of the dual console system during RALP training resulted in shortened operative time and reduced intra- and post-operative complications [24]. Interestingly these benefits did not translate to improvement in functional outcomes or biochemical recurrence with the dual console. The dual console systems are expensive training tools that are not available in every robotic institution. Given resource limitations, robotic training could be focused at institutions where the dual console is available. The assignment of resources and spread of training opportunities should be directed by national or international urological associations as part of formalised robotic surgical training programs.

Our study demonstrates the safety and efficacy of modular training supported by dual console support in RALP training. We acknowledge two main limitations in our study. The first is our focus on peri-operative and pathological outcomes only. We did not examine functional outcomes, owing to difficulties with data collection and the heterogeneous nature of functional data. While acknowledging this limitation, we provide a valuable assessment of the effect of trainee involvement in RALP by dual console on operative outcomes. Another limitation in our study is the contribution of a trainee to steps in specialist-lead cases. RALPs were classified as ‘trainee-lead’ if the trainee completed > 75% of the total operative steps. However, trainees completed between 0 and 38% of operative steps in consultant-lead cases. Trainee contributions to specialist-lead cases may contribute to the comparable pathological and operative outcomes between specialist-lead and trainee-lead cases demonstrated in our study. However, the majority of trainee contributions in specialist-lead cases are in the early steps of the case, of which a number are not highly technical. The small involvement of trainees in specialist-lead cases in our study reflects real-world training environments. These specialist-lead cases thereby serve as an appropriate comparison to examine the impact of operative progression of trainees on patient outcomes in surgical training institutions.

This study demonstrates that well-supervised trainees can produce comparable surgical and oncological outcomes in RALP to specialist-lead cases. In this era of increasing robotic surgery, learning curves for RALP and urological robotic surgeries can be initiated and improvised early in a surgical trainee’s career [23, 25]. Further, a formalised robotic curriculum should be created and incorporated as part of the Australian Surgical Education and Training Program. Such a modular curriculum may shorten the learning curve in robotic surgery without compromising patient outcomes [25].

## Conclusion

RALP can be performed by urology trainees with equivalent peri-operative and pathological outcomes when compared to operations performed by consultants. This is achieved by graded progression and close supervision from a dual console.

## Supplementary Information

Below is the link to the electronic supplementary material.

Supplementary file1 (DOCX 40 KB)

## Data Availability

No datasets were generated or analysed during the current study.
