# Supplementary material for: Comparing outcomes of robotic-assisted radical prostatectomy by specialists and trainees using a modular training approach
Source: J Robot Surg. 2025 May 13;19(1):215. doi: 10.1007/s11701-025-02277-6 (PMC12075022; doi:10.1007/s11701-025-02277-6)
Supplement: Supplementary file 1 — Supplementary file1 (DOCX 40 KB) [file 11701_2025_2277_MOESM1_ESM.docx]

Prostatectomy Worksheet

- G+H
- Plt
- Coags
- Anticoagulants
- LUTS
- ED
- Nerve spare plan
- LN plan
- MRI

Patient Name:

MRN: DOB:

Date of surgery:

Consultant:

Operating consultant:

**Patient Demographics**

Age: Weight: Height:

ASA grade: BMI:

Previous abdominal surgery: Antiplatelet/Anticoagulants:

Salvage case: Hb 0

DRE: PSA:

LUTS (IPSS): Erectile function (SHIM):

**Biopsy Results: (please attach biopsy results to this sheet)**

Date:

ISUP score: Max Core %:

Numbers of core positive: Total Number of cores taken:

Apex- Posterior

Apex- Anterior

Right

Left

Left

Right

Base- Posterior

Base- Anterior

**Imaging Results: (Please attach any reports)**

MRI Date/Location:

Prostate volume: PIRADS score:

MRI lesion: Lesion size: Evidence of T3 disease:

**Staging**

PSMA: Date:

Prostate Uptake: - T3 disease:

LN: Distant mets:

CT A-P:

Bone scan:

**Operative Information:**

Console Time:______________

Nerve spare type Right: _______________________ Left: ___________________________________

Intra-operative blood loss: _______________________ ml

Intra-operative complications/difficulties: ___________________________________________________________________________________________________________________________________________________________________________________________________________________________________________________________________________________________________________________________________________________________________________________________________________________________________________________________________________________________

- Call patient family

*Operative steps completed (tick applicable box):*

Date:

| # | Step | Completed by Trainee | |
| --- | --- | --- | --- |
| 1 | Positioning, port access and placement |  | |
| 2 | Retropubic space dissection, dropping of bladder |  | |
| 3 | Endopelvic fascia incision | Right | Left |
| 4 | Anterior bladder neck transection |  | |
| 5 | Posterior bladder neck transection |  | |
| 6 | Seminal vesical and vas dissection | Right | Left |
| 7 | Denonvilliers’ fascia incision, posterior dissection |  | |
| 8 | Lateral pedical dissection | Right | Left |
| 9 | Dorsal venous complex, puboprostatic ligaments |  | |
| 10 | Apical dissection and urethral division |  | |
| 11 | Rocco stitch |  | |
| 12 | Urethrovesical anastomosis |  | |
| 13 | Pelvic lymph node dissection | Right | Left |

Registrar/Trainee:

Consultant:
